# Multi-Sensor Based Online Attitude Estimation and Stability Measurement of Articulated Heavy Vehicles

**DOI:** 10.3390/s18010212

**Published:** 2018-01-13

**Authors:** Qingyuan Zhu, Chunsheng Xiao, Huosheng Hu, Yuanhui Liu, Jinjin Wu

**Affiliations:** 1 Department of Mechanical and Electrical Engineering, Xiamen University, Xiamen 361005, China; chunsheng.xiao@foxmail.com (C.X.); lyh0110@foxmail.com (Y.L.); wujinjinnice@foxmail.com (J.W.); 2 School of Computer Science & Electronic Engineering, University of Essex, Wivenhoe Park, Colchester CO4 3SQ, UK; hhu@essex.ac.uk

**Keywords:** articulated heavy vehicles, multi-sensor system, attitude and heading reference systems, rollover stability, vehicle safety

## Abstract

Articulated wheel loaders used in the construction industry are heavy vehicles and have poor stability and a high rate of accidents because of the unpredictable changes of their body posture, mass and centroid position in complex operation environments. This paper presents a novel distributed multi-sensor system for real-time attitude estimation and stability measurement of articulated wheel loaders to improve their safety and stability. Four attitude and heading reference systems (AHRS) are constructed using micro-electro-mechanical system (MEMS) sensors, and installed on the front body, rear body, rear axis and boom of an articulated wheel loader to detect its attitude. A complementary filtering algorithm is deployed for sensor data fusion in the system so that steady state margin angle (SSMA) can be measured in real time and used as the judge index of rollover stability. Experiments are conducted on a prototype wheel loader, and results show that the proposed multi-sensor system is able to detect potential unstable states of an articulated wheel loader in real-time and with high accuracy.

## 1. Introduction

**Figure 1:**
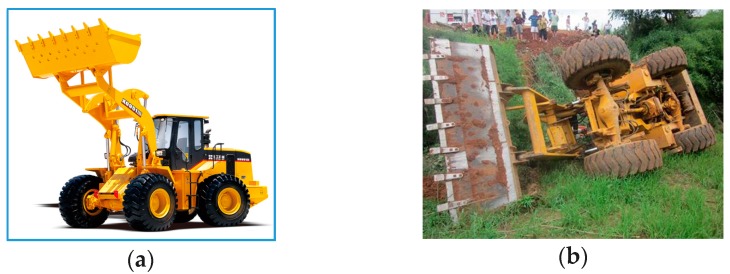
Articulated wheel loader and its rollover accident. (**a**) An articulated wheel loader [2]; (**b**) A rollover accident [3].

Articulated wheel loaders have multi body structure and are widely used in engineering construction sites, i.e., unstructured ground. They have an articulated steering mechanism that makes them adaptive to complex environments, and have a reduced turning radius in narrow fields, as well as an improved off-road performance [1]. Figure 1a shows that a wheel loader is equipped with a powerful bucket for a wide range of loading tasks, in which the bucket could be replaced by other types of working devices for different tasks.

However, articulated wheel loaders have poor stability and high rate of accidents, which is normally caused by dynamic and unpredictable changes of their body posture, mass and centroid position in a complex operation environment. Figure 1b shows the rollover accident of a wheel loader, which seriously threatens the safety of the driver. Therefore, effective protective measures must be taken to prevent rollover accidents, and many research studies have been conducted recently. The active safety control is one of the most direct and effective methods [4,5], which was realized by the prediction and judgment of the lateral buckling state.

Up to now, many researchers used geometric theory to characterize vehicle stability, and constructed an instability indicator using the relationship among the weight center of the vehicle, the tire grounding location and the position of the resultant force vector [6]. This force-angle based instability indicator was widely used in a multi-legged robot on the rugged road [7]. The key factor of real-time measuring of this kind of index is to measure inertial force net, as well as to estimate the vehicle centroid position and the location of the tire grounding points.

Nalecz et al. proposed the method of rollover prevention energy reserves (RPER) from the point of energy, researching all kinds of lateral tilting situations caused by stumbling [8,9]. The energy was chosen as a rollover threshold to enhance the vehicle’s weight center to the rollover point. Chen et al. optimized this energy based method by measuring kinetic parameters, such as the vehicle roll angle velocity, yaw velocity, lateral acceleration and vehicle driving speed to accurately estimate the roll angle of the vehicle [10]. Moreover, they used a linear combination of the roll energy and the rate of roll energy to characterize lateral stability of plate. 

In general, the tire-ground contact force could directly represent the stability of the vehicle, which was used by many researchers to study vehicle stability. At the same time, other methods were also used for vehicle stability analyses, such as the tire-ground contact angle [11], the tire-ground contact torque [12], and the lateral load transfer rate (LTR) [13]. However, since direct measurements of the tire-ground contact force were difficult in practice, the indirect method was widely adopted to calculate the dynamic load of the vehicle. Kamnik et al. proposed the use of a sensory system to estimate the lateral load transfer, which was implemented on a vehicle with multi-axis accelerometers and angle rate sensors [14]. Miege et al. proposed a real-time method for computing the lateral load transfer rate, which was based on measuring the lateral acceleration and the lateral tilt angle [15].

As the reliability and scope of single index are limited, some combined indexes have been deployed in the rollover instability indicators to truly reflect the vehicle state [16,17,18]. However, these research studies were based on normal road vehicles that have relatively fixed structure, mass and center of weight. Their research results cannot be directly applied to articulated wheel loaders that have multibody structure, random changes of the mass center, as well as operated on uneven road surfaces. Therefore, we need a more appropriate index and its measurement system for the articulated wheel loader.

Li et al. pointed out that the lateral acceleration, angular velocity and the slope angle were the main factors that led to the instability of articulated steering vehicles [19]. Then, they got the dynamic stability index of the articulated steering vehicle by installing sensors at the rotating joints. Furthermore, the displacement, angle and inertial measurement were used to calculate the dynamic stability index in real-time. 

Moreover, the current rollover instability indicator for variable structure vehicles has many parameters to be measured and the sensor installation could be very difficult. The advantages and disadvantages of these indicators are listed in Table 1. Therefore, this paper proposes the use of SSMA as a new instability prediction index to monitor lateral stability of an articulated wheel loader whose special structure and lateral tilting process have been taken into consideration. Based on our previous work in [20], we use an on-line measurement system with four AHRS modules to monitor the attitude and lateral stability of an articulated wheel loader. Compared with other measurement systems, the proposed method is suitable to multi-body vehicles, which is much cheaper and easy to install. 

The rest of the paper is organized as follows. Section 2 describes the construction of a multi-sensor system for the online measurement of SSMA. Section 3 introduces the SSMA measurement using AHRS and the calibration of the proposed measurement system. Then, experiment results and analysis are presented in Section 4 to verify the feasibility and performance of the proposed online measurement approach. Finally, a brief summary and future research direction are given in Section 5.

## 2. Measurement System Construction 

To reduce the risk of conducting experiments on a real articulated wheel loader, we have built a physical prototype of an articulated wheel loader to carry out experiments in our laboratory, which has a ratio of 1:5 in size with respect to XG958 wheel loader made by Xiamen XGMA Machinery Co., Ltd. (Xiamen, China) The dimensions and specifications of the physical prototype are given in Table 2. 

**Figure 2:**
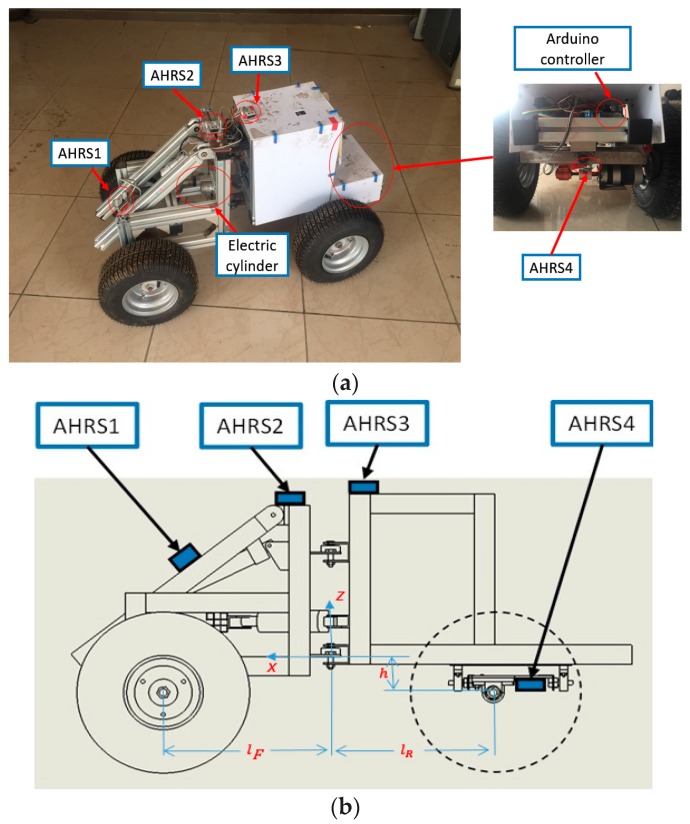
Scale prototype model for experiment and its sensor installation. (**a**) Scale down prototype model the actual installation location of four AHRS; (**b**) Sensor location and the location of some parameters.

As shown in Figure 2a, the steering section is driven by the electric cylinder that is installed between the front and the rear body. An Arduino controller was installed at the back of the prototype to control the speed and articulated angel. Figure 2b shows its hardware structure and the installation of the measurement system, as well as the location of some parameters. As can be seen, four AHRS modules are installed on the model’s swing arm, front body, rear body and rear axle, namely AHRS1, AHRS2, AHRS3, and AHRS4. Every AHRS provides an attitude and heading reference system used to measure the attitude quaternion and acceleration of the wheel loader. The measured data is then transmitted to the main processor module via a data cable. The main processor module will then transmit the information to the converged data center for subsequent processing and analysis.

**Figure 3:**
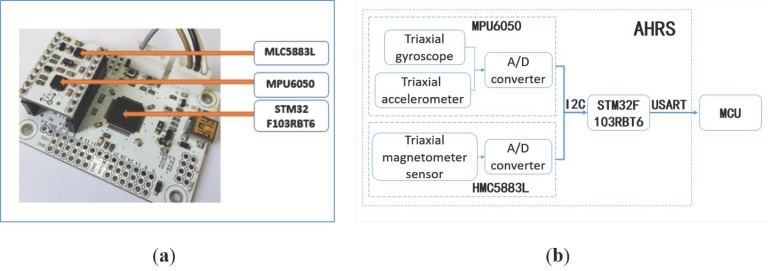
The hardware structure of each AHRS. (**a**) Hardware; (**b**) Configuration.

Figure 3a shows the AHRS hardware and Figure 3b shows how the AHRS system is configured. Each AHRS consists of a six-axis motion processing sensor module MPU6050 and a magnetic sensor module HMC5883L, which can be used to measure a rigid body independently. MPU6050 integrates a tri-axial MEMS gyroscope with a measurement range of ±500°/s and tri-axial MEMS accelerometer with a measurement range of ±4 g. It also has a 16-bit A/D converter to output six-axis information through an I2C interface and a digital temperature sensor for temperature compensation. HMC5883L is a magnetic sensor chip with an I2C interface, based on Hallowell anisotropic magnetoresistance technology. It has high sensitivity and linearity with a measurement range of ±8 Gaussian.

We use a F103RBT6 STM32 microcontroller unit (MCU) to read data from the two sensor modules mentioned above through an I2C interface, and output the fused data via a RS232 interface. The overall size of each AHRS module is 45 × 60 × 20 mm. Among the four AHRS, AHRS3 is mounted on the rear body accurately, with its Y axis parallel to the rear body chassis and point to vehicle forward direction, and its Z axis perpendicular to the vehicle chassis. It is used to represent the posture of the body reference frame. The other three AHRS modules are used to calculate the attitude change of the prototype wheel loader with respect to AHRS3, so they are simply installed on the corresponding components.

**Figure 4:**
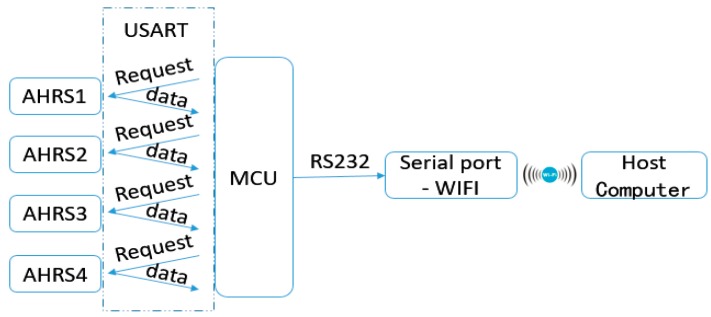
The configuration of the proposed multi-sensor system.

**Figure 5:**
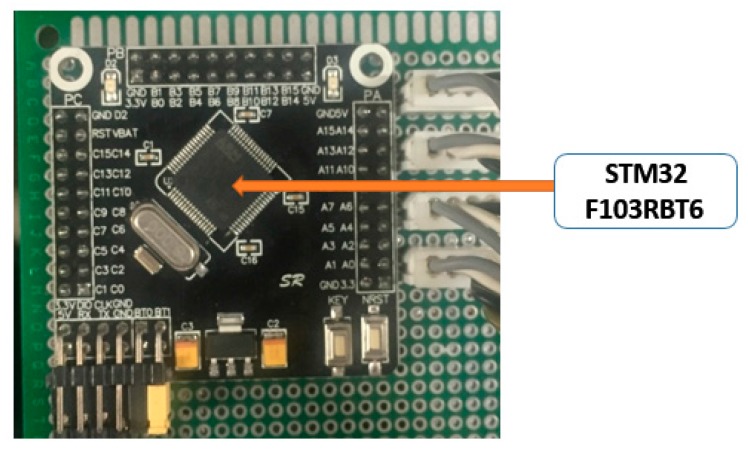
The hardware structure of a centralized MCU.

As we know, a multi-sensor system has a distributed structure whose advantages include fast calculation, good reliability and low communication bandwidth [21,22]. Figure 4 shows our multi-sensor system which consists of the four AHRS, the MCU and the host computer. Each AHRS will collect data from its gyroscope, accelerometer and magnetic sensor through its I2C bus at a frequency of 400 Hz. The data obtained from each individual AHRS is processed within its own STM32 32-bit Acorn RISC Machine (ARM) Cortex MCU, and then sent to a centralized MCU shown in Figure 5 to calculate the attitude quaternion and acceleration of the wheel loader. The results are then sent to the fusion center for analysis and storage via wireless WIFI communication.

Since the sampling rate and time of each sensor are different, the synchronization of the data sampling in a multi-sensor system has a big impact on the results of data processing. We have chosen the data polling method here to realize the approximate synchronous sampling as the response frequency of the system is low.

A centralized STM32 MCU is used as the main processor module to collect data from four AHRS modules. It firstly sends a falling edge signal to pin PA13 of AHRS1 to collect its latest collected data through its USART serial port. Similarly, the main MCU sends a falling edge signal to the next AHRS until the last one, i.e., AHRS4. As only one AHRS could send data to the main processor module at each moment, the main processor module can use one USART serial port to receive and send four sets of data from and to four AHRS modules in a cycle.

It should be noted that the sampling frequency of the main processor module is 20 Hz, i.e., the time interval between every two cycles is 50 ms. Therefore, the data from the four AHRS modules can be collected in 50 ms by the main processor module to achieve approximate synchronous sampling.

## 3. SSMA Measurement Based on AHRS

### 3.1. SSMA Definition

**Figure 6:**
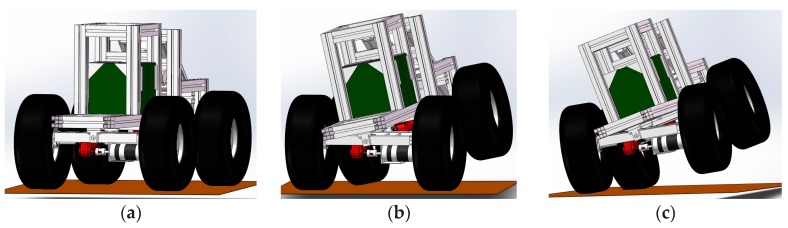
The lateral tilting process. (**a**) Steady state; (**b**) First stage unbalancing; (**c**) Second stage unbalancing.

Since a wheel loader has an articulated body structure, its body swing angle and the rear axle will change dynamically during operation, as well as its bucket location and loading. This leads to the random change of its body posture, mass and centroid position. Therefore, its lateral tilting process is more complex than normal road vehicles as shown in Figure 6. Figure 6a shows the steady state of the wheel loader. When the lateral force applied to the wheel loader is large enough, one tire will leave the ground, which is called the first stage unbalancing, as shown in Figure 6b. At this time, the other three wheels are contacting with the road, the wheel loader is in a critical steady state. After the first stage unbalancing occurs, another wheel may leave the ground if the lateral force is big enough, as shown in Figure 6c. This is called the second stage unbalancing.

**Figure 7:**
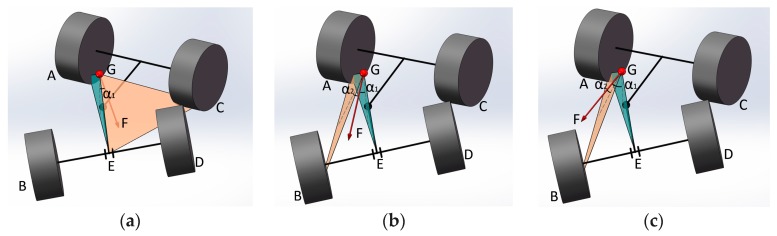
The relative location map of resultant force vector and stabilizing plane for an articulated wheel loader. (**a**) Steady state; (**b**) First stage unbalancing; (**c**) 2nd stage unbalancing.

Table 3 defines the relevant symbols used in this paper, in which the plane △GAE and the plane △GCE are called the stable surface for the first stage unbalancing. The plane △GAB and the plane △GCD are called the stable surface for the second stage unbalancing. As shown in Figure 7a, when the wheel loader is at a stable state, $\vec{F}$ must be between the plane △GAE and △GCE, $\alpha_{1}>0$, $\alpha_{2}>0$; When the force vector $\vec{F}$ is outside the plane △GAE (or △GCE), it will provide the wheel loader a torque to flip around the lateral tilting axis, leading to the first stage unbalancing, at this time $\alpha_{1}<0$, $\alpha_{2}>0$, as shown in Figure 7b; if the resultant force is outside the plane △GAB (or △GCD), as shown in Figure 7c, the wheel loader will flip around the second stage unbalancing axis AB (or CD), eventually leading to the lateral tilting accident, at this time $\alpha_{1}<0$, $\alpha_{2}<0$.

Therefore, the resultant force $\vec{F}$ and the position of the stable surface affect the lateral stability of the wheel loader. The angles, $\alpha_{1}$ and $\alpha_{2},$ are the SSMA for the first and second stage unbalancing, respectively. They are used as the indexes to characterize the rollover danger of a wheel loader, and can be described as follows [20]:

(1) {n1→=p 0Gp 0A→×p 0Gp 0E→n2→=p 0Gp 0B→×p 0Gp 0A→αi=arcsin(F→×ni→|F→|×|ni→|)−90°, i=1, 2 

where p 0A ~ p 0D indicate the different wheel grounding points in the body coordinate system. p 0G is center of weight point and p 0E is the panel point of rear body and rear axle in the body coordinate system. $\vec{n_{1}}$ is the normal vector of plane △GAE and $\vec{n_{2}}$ is the normal vector of plane △GAB. 

### 3.2. Measurement Principle

SSMA reflects the status of the wheel loader itself, which enables the wheel loader’s own state to be used to determine its lateral stability without considering the external cause of lateral tilting. It not only describes the change of lateral stability caused by resultant force vector, but also describes the change of lateral stability due to the change of the center-of-mass’s position caused by the flexible structure of wheel loaders.

The key factor for measuring SSMA is to construct a stable surface and measure the resultant force vector. The calculation of the resultant force vector is related to the actual operating condition of the wheel loader and is reflected in the acceleration directly as follows.

(2) $$\vec{F}=(\overset{5}{\underset{i=1}{\sum }}M_{i})\vec{a}$$

where $M_{1}$, $\;M_{2}$, $\;M_{3}$, $\;M_{4}$ are the mass of swing arm, front body, rear body and rear axle, respectively. $\;M_{5}$ indicates the mass of the load, $\vec{a}$ indicates the three-axis acceleration of the loader which can be measured by the AHRS which is mounted on rear body.

Constructing stable surfaces is to get the coordinates of the wheel grounding point (A,B,C,D), the center of weight point (G) and the panel point of rear body and rear axle (E). The position of point E in in the body coordinate system (p 0E) is constant and its value is related to the structural parameters of the wheel loader (they are shown in Table 2). The position of point A (p 0A) and point B (p 0B) in the body coordinate system can be obtained by the following formulas.

(3) p 0A=R20×(p 0O2+p 2A)

(4) R20=[cosγ−sinγ0sinγcosγ0001]

(5) p 0B=R40×(p 0O4+p 4B)

(6) R40=[cosθ0sinθ010−sinθ0cosθ]

where γ is the chassis steering angle and θ is rear axle swing angle; p 0O2 and p 0O4 indicate the position of AHRS2 and AHRS4 in the body coordinate system at the initial moment, respectively; p 2A and p 4B indicate the wheel grounding points in the carrier coordinate system of AHRS2 and AHRS4.

Then, the center of weight point in the body coordinate system (p 0G) can be calculated by the theorem on moment of resultant force:

(7) p 0G=∑i=15Mip 0Gi∑i=15Mi

where p 0Gi (i=1, 2, 3, 4) is the center position of part i in the body coordinate system; p 0G5 indicates the position of the load center in the body coordinate system; p 0Gi can be calculated by coordinate transformation formula.

(8) p 0G1=R20×(p 0o2+p 2o1+R12×p 1G1)

(9) p 0G2=R20×p 0o2

(10) p 0G3=p 0o3

(11) p 0G4=R40×p 0o4

(12) p 0G5=R20×(p 0o2+p 2o1+R12×p 1G5)

(13) R12=[1000cosβ−sinβ0sinβcosβ]

where β is the swing arm lifting angle. p 2o1 is the position of AHRS1 in the carrier coordinate system of AHRS2, p 1G1 and p 1G5 are the center position of part one and load in the carrier coordinate system of AHRS1 at the initial moment.

**Figure 8:**
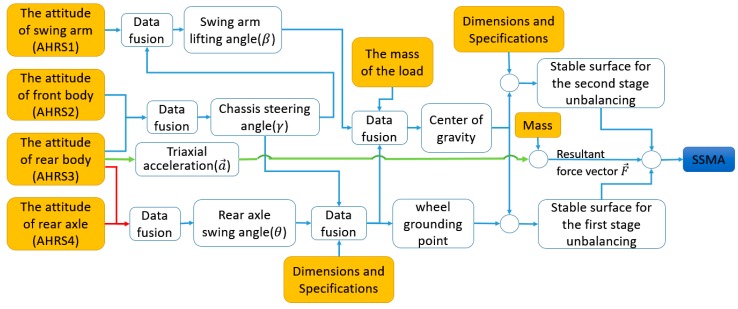
SSMA measuring principle.

Figure 8 shows the basic principle for on-line detection to measure SSMA. Four AHRS are used here (their installation position is shown in Figure 2) to calculate the relative attitude for the reason that a wheel loader can be modeled as four articulated rigid bodies. The rear axle swinging angle is calculated by the relative change between AHRS3 and AHRS4. The swing arm lifting angle and chassis steering angle are calculated by the relative change among AHRS1, AHRS2 and AHRS3. The triaxial acceleration of AHRS3 is used to approximate the acceleration of the loader. Combining these information with dimensions and specifications of loaders and load mass, we can calculate the angle between the stable surface and the resultant force vector which is SSMA. The algorithm for SSMA is shown in Appendix A.

### 3.3. Measurement Accuracy Calibration of AHRS

#### 3.3.1. Complementary Filtering Algorithm

The data from a three-axis gyroscope, a three-axis accelerometer and a three-axis magnetic sensor within each AHRS is noisy. A data fusion method is required to deal with the noisy data [23]. At present, Kalman filter is the most commonly used data fusion method for attitude estimation. However, its implementation needs a precise system model and noise characteristics, as well as a high-performance computer [24]. 

On the other hand, a complementary filter is a kind of data fusion method and can be used in a multi-sensor system to improve the accuracy and stability of the system. In our AHRS, the dynamic performance of the gyroscope is superior, and its noise is mainly distributed in the low frequency band, so the dynamic changes of the attitude angle can be very accurate. In contrast, the static performance of the accelerometer and the magnetic sensor is superior, and their noise is mainly distributed in the high frequency band. 

**Figure 9:**
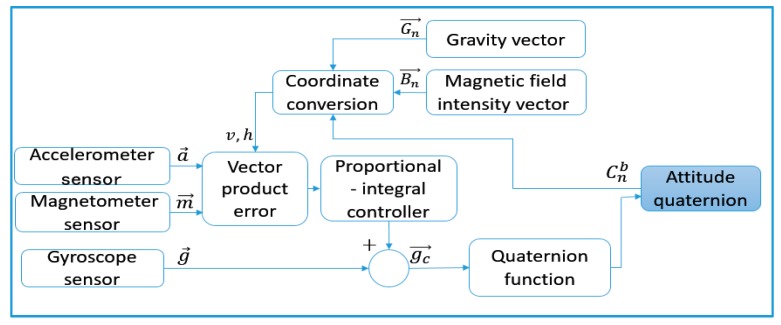
Calculation process of complementary filtering algorithm.

Therefore, we use high pass filtering to process the gyroscope integral results and low pass filtering to process accelerometer and magnetic sensor data. In this way, gyro drift and its attitude angular deviation caused by numerical integration can be effectively compensated so that the accurate and stable attitude angle can be obtained [25]. Figure 9 shows the calculation process of the complementary filter algorithm. 

In the geographic coordinate system, the weight vector is:

(14) $$G_{n}=\left[0,0,g\right]$$

The strength of the local magnetic field with different longitude and latitude coordinates are different, so the geomagnetic field cannot be expressed by constant value like acceleration in the geographical coordinate system. By the definition of the geographic coordinate system, the component of the geomagnetic field in the horizontal plane coincides with the X axis of the geographic coordinate system, so the geomagnetic field vector could be expressed as $B_{n}$ in the geographical coordinate system.

(15) $$B_{n}=\left[b_{x},0,b_{z}\right]$$

While in the AHRS Carrier coordinate system, the acceleration is expressed as:

(16) $$a=\left[a_{x},a_{y},a_{z}\right]$$

The geomagnetic field strength is expressed as:

(17) $$m=\left[m_{x},m_{y},m_{z}\right]$$

If the Carrier coordinate system is only affected by weight instead of the geomagnetic field, the vectors $G_{n}$ and $B_{n}$ in the reference coordinate system and the vectors *a* and *m* in the Carrier coordinate system represent the weight vector and the geomagnetic field vector respectively. We construct the rotation matrix $C_{n}^{b}$ using the attitude quaternion measured and calculated by AHRS, and then convert $G_{n}$ and $B_{n}$ to the Carrier coordinate system:

(18) $$v={\;C}_{n}^{b}⊗G_{n}$$

(19) $$h=C_{n}^{b}⊗B_{n}$$

Theoretically, v and h should be the same as the measured value a and m in the Carrier coordinate system. However, if the measurement attitude quaternion is not accurate, there would be error in the rotation matrix $C_{n}^{b}$; v and h would not be the same as a and m.

(20) $$e_{1}=a⊗v$$

(21) $$e_{2}=m⊗h$$

The error $e_{1}$ and $e_{2}$ can be obtained by vector product operation among v, h, a and m, then accurate and stable attitude quaternion can be obtained after the errors corrected by PI controller. The complementary filtering algorithm is detailed in Appendix B.

#### 3.3.2. AHRS Calibration Results

Similar to the work in [26], we carry out a number of static and dynamic tests to analyze and verify the static and dynamic measurement accuracy of AHRS in order to verify the reliability of the proposed multi-sensor system and the complementary filtering fusion algorithm. The static test process is to keep AHRS static and then continuously collects data for 1 h after 10 min warm-up. 

**Figure 10:**
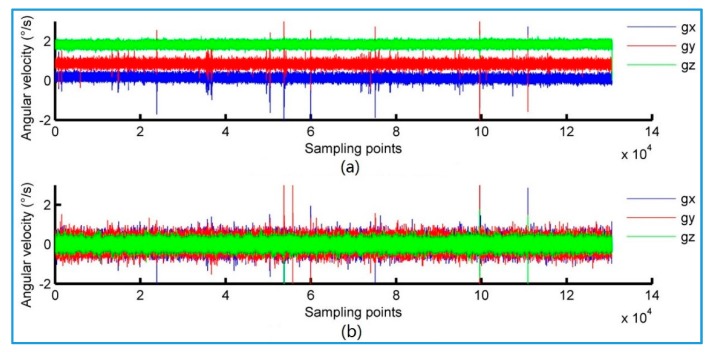
Contrast diagram before and after complementary filtering. (**a**) Gyro original angular velocity; (**b**) Filtered angular velocity.

Figure 10 shows the original angular velocity data output from gyro and the angular velocity data calculated by the complementary filter algorithm. The original data output from gyro has random drift error, and the attitude information calculated by using the original data gyroscope data will generate random integral errors. In contrast, the filtered angular velocity has no drift error, which has Gaussian white noise with zero mean. The automatic compensation of the gyro random drift error is achieved. Therefore, the attitude data obtained by the filtered angular velocity is relatively stable.

**Figure 11:**
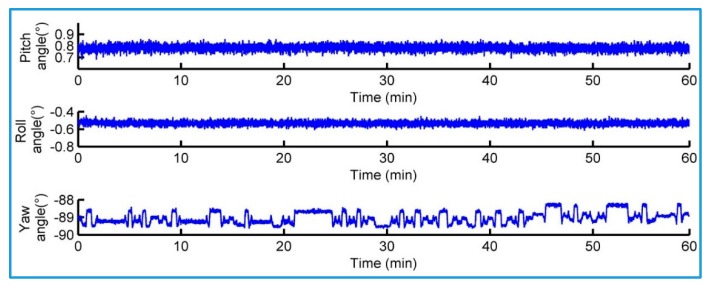
Attitude angle data AHRS output in static state.

Figure 11 shows the attitude angle data that is output by AHRS under its static state. As can be seen, there is no random integral error. The standard deviations of the pitch angle, the lateral angle and the yaw angle are 0.027°, 0.025° and 0.426°, respectively. 

**Figure 12:**
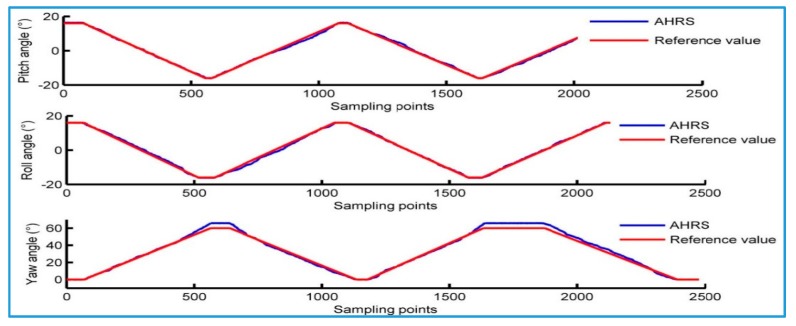
Comparison between output of AHRS and reference values.

The dynamic testing process is to install the AHRS on a six-axis platform with a high precision (model: PI H-840). The minimum increment of the platform rotation around the X, Y and Z axes is 5 μ radian, which has high motion precision. During the test, the platform is rotating along one axis at a constant speed, and form a zigzag curve shown in Figure 12. Then, the measurement data of AHRS and the reference value of the actual motion are compared to evaluate the dynamic measurement error of AHRS. Since the platform’s movement accuracy is high, we use the angle change curve as the reference value to represent the actual movement situation, which is similar to the approaches conducted in [27,28].

**Figure 13:**
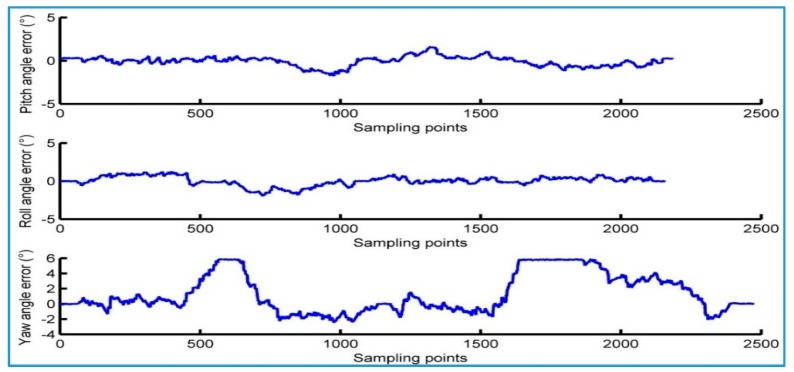
Error of AHRS in dynamic test.

Figure 13 shows the difference between the AHRS output and the reference value of the actual motion. It is used to represent the dynamic error of the attitude angle that AHRS outputs. The root mean square of the dynamic error for its pitch angle, roll angle and yaw angle is 0.577°, 0.634°and 2.513°, respectively.

## 4. Lateral Tilting Test and Data Analysis Results

### 4.1. Static Test

**Figure 14:**
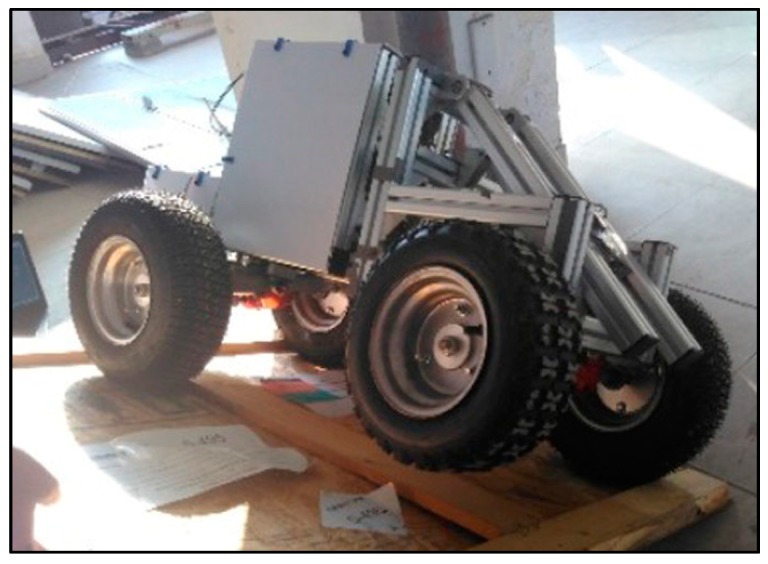
Static rollover stability test site.

Figure 14 shows the test site in our laboratory. During the static rollover stability test, the prototype wheel loader is placed on a rotatable test bench. We rotated the test bench at an average speed of 0.02 rad/s until the inner front wheel of the wheel loader off the test bench. Then SSMA and the limiting slope angle of the first stage unbalancing are measured.

**Figure 15:**
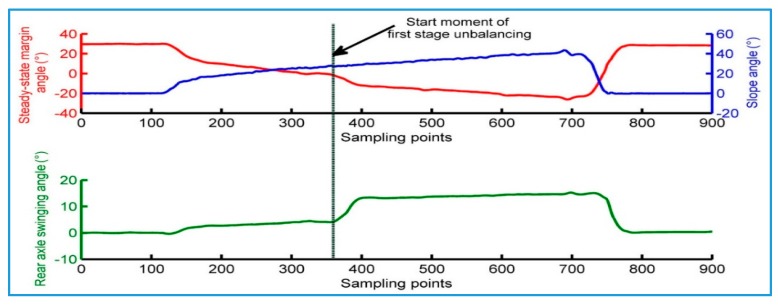
Static rollover stability test data.

Figure 15 shows the data in the process of the static rollover stability test. As can be seen, the SSMA data is decreasing when the lateral slope angle is increased. Before the first stage unbalancing, the tire deformation changes due to the lateral offset of the prototype wheel loader. The deformation of the tire near the bottom side increases and the deformation of the other side decreases, which causes a small change in the swinging angle of its rear axle. The first stage of unbalancing occurs when the sampling points is about 360. The frame rotates around the rear axle, and causes a large change in the swinging angle of the rear axle. This continues until the frame stop block is in contact with the rear axle, and the swing angle reaches a maximum value of 11.5°. The change tendency of the curve can prove that the measured SSMA can accurately reflect that the lateral stability of the prototype wheel loader varies with the increase of the lateral slope angle. 

The vertical line in Figure 15 is the start moment of the first stage of imbalance, which is chosen at the moment when the rear axle swinging angle begins to change significantly. The SSMA and slope angles at this moment are similar to the ones when the first stage unbalancing occurs. 

**Figure 16:**
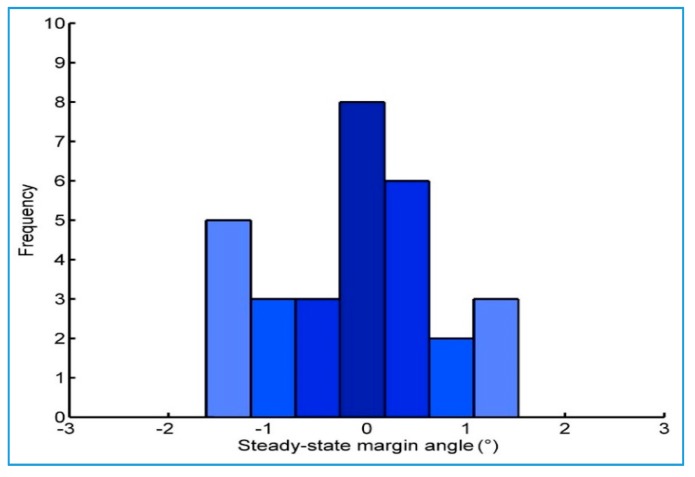
SSMA distribution map (30 groups of static tests).

We conducted 30 groups of static rollover stability tests. Figure 16 shows the distribution of SSMA when first stage unbalancing occurs, in which the average value is −0.1028°, the standard deviation is 0.862° and the average value of the limited slope angle is 27.585°. Theoretically, SSMA is 0 ± 1.5° when the first stage unbalancing occurs, which matches the test results. Therefore, the measured SSMA can accurately characterize the lateral stability of the prototype wheel loader in the static case.

### 4.2. Dynamic Test

**Figure 17:**
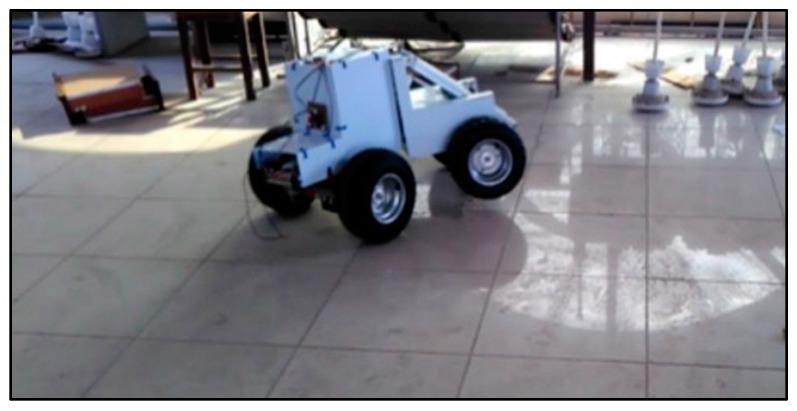
Steady-state rotation test site.

In order to obtain more stable dynamic data, we conducted dynamic tests to verify if SSMA could accurately characterize the lateral stability of a wheel loader. It was based on the basic principle in steady-state circular test procedures [29]. During the test, the prototype wheel loader implemented uniform circular motions at a fixed hinge steering angle. We obtained different lateral accelerations by changing its forward speeds, thereby changing the lateral stability of the vehicle. Figure 17 shows the test site in our laboratory.

**Figure 18:**
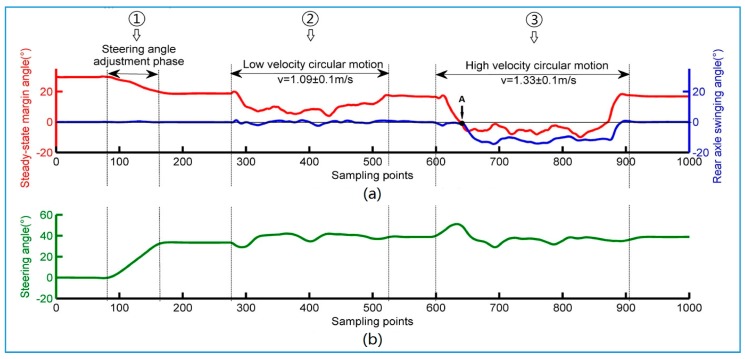
Uniform circular motion test data. (**a**) Steady-state margin angle and rear axle swinging angle curve; (**b**) Steering angle curve.

Figure 18a shows the change curve of steady state margin and the swing angle of the rear axle when the wheel loader is in a uniform circular motion with a steering angle of 35°. The uniform circular motion is divided into three phases: the steering angle adjustment phase, the low speed circular motion phase and the high-speed circular motion phase. Figure 18b shows that the steering angle is relatively stable and always floating in a small range during the entire circular motion.

The initial stage of the curve is the process of adjusting the steering angle with a zero speed. It can be seen that SSMA and the lateral stability of the wheel loader decreases with the increase of the steering angle. When the steering angle reaches 35° at the beginning of phase ②, the wheel loader speeds up to 1.09 ± 0.1 m/s and begins to do low speed circular motion. During this period, SSMA continues to decrease but is still greater than 0°, and the rear axle swinging angle has not changed greatly. After a low speed circular motion at the beginning of phase ③, the wheel loader speeds up to 1.33 ± 0.1 m/s at a high-speed circular motion.

In the initial stage of high speed circular motion, front and rear bodies start to rollover while the SSMA decreases; at point A in Figure 18a, SSMA is reduced to 0°. The inside front wheel of the wheel loader leaves the ground, and the wheel loader starts the first stage of unbalancing. Then, the front and rear bodies keep rolling over until the swinging angle reaches a maximum of 11.5°. Eventually, the wheel loader goes back to stable state through constant deceleration. The experiments show that the measured SSMA can accurately reflect the dynamic stability of the wheel loader in uniform circular motions.

## 5. Conclusions

In this paper, a multi-sensor system has been designed for real-time attitude estimation and stability measurement of articulated wheel loaders. Each sensor module, AHRS, consists of a six-axis motion processing sensor modules MPU6050 (a tri-axial MEMS gyroscope and a tri-axial MEMS accelerometer) and a magnetic sensor module HMC5883L. Four AHRS modules have been deployed to measure SSMA online, which has the advantages of real-time performance, easy installation and less parameters to be measured. Then, SSMA is used for comprehensive characterization of the lateral stability of an articulated wheel loader. The lateral tilting experiments were conducted on a physical prototype wheel loader. Experimental results proved that the SSMA obtained by the established online measuring system can accurately represent lateral stability of wheel loader under static and dynamic conditions.

During the real operation of an articulated wheel loader, its bucket may have different loading weight and position, which poses a challenge to its stability. Our future research will investigate the measurement of such loading changes under real working conditions. Furthermore, an active control system will be developed to adjust and control the unstable state of an articulated wheel loader to prevent potential rollover accidents.

## Figures and Tables

**Table 1:** Evaluation of various instability predictors indicators.

Principle of Indicators	Advantages	Disadvantages
Geometric principle	Simple principle	Ignore the changes in the height of the vehicle center of weight and the dynamic performance of the vehicle on its stability
Rollover energy	For tripping rollover	Ignore the various aspects of energy consumption in the rollover process
Contact force between tire and ground	Intuitively represent the stability of the vehicle	Tire ground contact force is difficult to accurately measure
Static threshold	Convenient and direct accurate	The warning performance is poor
Combination indicators	More reasonable and comprehensive represent of the vehicle rollover stability	Too many parameters need to be detected

**Table 2:** Dimensions and specifications.

Parameter	Explanation	Value
m(kg)	total mass of the prototype	56.1
$m_{F}\left(kg\right)$	front body mass	16.4
$m_{R}\left(kg\right)$	rear body mass	26.4
$m_{B}\left(kg\right)$	rear axle mass	13.3
$I_{\mathrm{XXF}},I_{\mathrm{YYF}},I_{\mathrm{ZZF}}\left(kg\;m^{2}\right)$	moment of inertia of front body	0.642,1.57,1.68
$I_{\mathrm{XXR}},I_{\mathrm{YYR}},I_{\mathrm{ZZR}}\left(kg\;m^{2}\right)$	moment of inertia of rear body	0.765,1.79,1.25
$I_{\mathrm{XXB}},I_{\mathrm{YYB}},I_{\mathrm{ZZB}}\left(kg\;m^{2}\right)$	moment of inertia of rear axle	0.413,1.58,1.89
$W_{F}\left(X_{F},Y_{F},Z_{F}\right)\left(m\right)$	center of weight of front body	0.26,0,0.005
$W_{F}\left(X_{R},Y_{R},Z_{R}\right)\left(m\right)$	center of weight of rear body	−0.19,0,0.14
$W_{F}\left(X_{B},Y_{B},Z_{B}\right)\left(m\right)$	center of weight of rear axle	−0.33,0,−0.04
B(m)	wheel track	0.45
$l_{F}\left(m\right)$	distance between pivot joint and the center of the front axle	0.32
$l_{R}\left(m\right)$	distance between pivot joint and the center of the rear axle	0.32
R(m)	tire radius	0.15
h(m)	distance between rear axle shaft and rear body longitude axis	0.02
$K_{\alpha }\left(N/{}^{\circ}\right)$	cornering stiffness of tire	81,600
$K_{V}\left(N/m\right)$	vertical stiffness of tire	20,000
$C_{V}\left(N\;s/m\right)$	vertical damping of tire	5000

**Table 3:** Symbol definition.

Parameter	Define
G	center of weight point
$\vec{F}$	the resultant force of weight and inertia force of the loader that acts on center of weight point G
A	left front wheel grounding point
B	left rear wheel grounding point
C	right front wheel grounding point
D	right rear wheel grounding point
E	the panel point of rear body and rear axle
$\alpha_{1}$	the angle between $\vec{F}$ and the plane △GAE (or △GCE)
$\alpha_{2}$	the angle between $\vec{F}$ and the plane △GAB (or △GCD)

## Appendix A

**Algorithm: The Algorithm for SSMA Calculation**
//The data acquisition algorithm is used to extract all sensor data; Generate Date List, Status list; Get all the date points $\;p_{i}\left(g,a,m,q\right)$, and put them in the Date List; Get all the status values $a_{i}$, and put them in the Status list; $K=\left(p_{1},\;p_{2},p_{3},p_{4}\right)$; i=1, 2, 3, 4; **For** *j* = 0 to Date List.num **do** Start the timer; Send $p_{i}$ to; **If** i=4, wait until the time end and get back; end **If**; Update the K; End //The calculation for SSMA Generate Date List, Status list; Get all the date points K, and put them in the Date List; Get the status value $K_{0}$ in K, and put it in the Status list; Put the default status value m in the Status list; **If** $K_{0}=m$, decode the $p_{i}$, and save it to the queue, save the initial data K; **If** i=1, 2, get $p_{i}$ to calculate the angel of movable arm, and save it to Date List; **If** i=2, 3, get $p_{i}$ to calculate the steering angel, and save it to Date List; **If** i=3, 4, get $p_{i}$ to calculate the swing angel, and save it to Date List; Get all them out including $p_{3}$ to calculate SSMA, and save it to Date List; End **If** Update the K, Update SSMA; End.

## Appendix B

**Algorithm: The Algorithm for Complementary Filtering**
//Complementary algorithm; Generate Date List, Status list; Get all the date points $a_{i}\left(ax,ay,az\right),\;g_{i}\left(gx,\;gy,\;gz\right),\;m_{i}\left(mx,my,mz\right)$, and put them in the Date List; Get all the status values , and put them in the Status list; **For** *j* = 0 to Date List.num **do** Get the $g_{i}$ out to calculate $\theta_{g}$; **If** t=kT; β=kβ, α=(N−k)α; Calculate$\;Qi=\alpha \theta_{g}+\beta \theta_{a}$; End** If**.

## References

[1] Rehnberg A. (2008). Vehicle Dynamic AAnalysis of Wheel Loaders with Suspended Axles. Ph.D. Thesis.

[2] http://www.qihuiwang.com/product/q33310/157848.html.

[3] http://119.china.com.cn/qxjy/txt/2012-06/27/content_5115836.htm.

[4] Palkovics L., Semsey A., Gerum E. (1999). Roll-over prevention system for commercial vehicles–additional sensorless function of the electronic brake system. Veh. Syst. Dyn..

[5] Chen B.-C., Peng H. (2001). Differential-braking-based rollover prevention for sport utility vehicles with human-in-the-loop evaluations. Veh. Syst. Dyn..

[6] Mcghee R.B., Iswandhi G.I. (1979). Adaptive locomotion of a multi-legged robot over rough terrain. IEEE Trans. Syst. Man Cybern..

[7] Papadopoulos E., Rey D.A. (2000). The force-angle measure of tipover stability margin for mobile manipulators. Veh. Syst. Dyn..

[8] Nalecz A.G., Bindemann A.C., Bare C. (1988). Sensitivity analysis of vehicle tripped rollover model.

[9] Nalecz A.G. (1989). Influence of vehicle and roadway factors on the dynamics of tripped rollover. Tech. Phys. Lett..

[10] Moshchuk N.K., Chen S.K., Chen C.F. (2010). Roll Stability Indicator for Vehicle Rollover Control. U.S. Patent.

[11] Iagnemma K., Rzepniewski A., Dubowsky S., Schenker P. (2003). Control of robotic vehicles with actively articulated suspensions in rough terrain. Autonom. Robot..

[12] Peter S.C., Iagnemma K. (2009). Stability measurement of high-speed vehicles. Veh. Syst. Dyn..

[13] Rakheja S., Piché A. (1990). Development of directional stability criteria for an early warning safety device. Sea Pap..

[14] Kamnik R., Boettiger F., Hunt K. (2003). Roll dynamics and lateral load transfer estimation in articulated heavy freight vehicles. Proc. Inst. Mech. Eng. Part D J. Automob. Eng..

[15] Miege A.J.P., Cebon D. (2005). Active roll control of an experimental articulated vehicle. Proc. Inst. Mech. Eng. Part D J. Automob. Eng..

[16] Jin Z.L., Weng J.S., Hu H.Y. (2007). Rollover stability of a vehicle during critical driving manoeuvres. Proc. Inst. Mech. Eng. Part D J. Automob. Eng..

[17] Larish C., Piyabongkarn D., Tsourapas V., Rajamani R. (2013). A new predictive lateral load transfer ratio for rollover prevention systems. IEEE Trans. Veh. Technol..

[18] Jin Z., Zhang L., Zhang J., Khajepour A. (2016). Stability and optimized H_∞_ control of tripped and untripped vehicle rollover. Veh. Syst. Dyn..

[19] Li X., Wu Y., Zhou W., Yao Z. (2016). Study on roll instability mechanism and stability index of articulated steering vehicles. Math. Probl. Engeerg.

[20] Zhu Q., Yi J., Chen H., Wen C., Hu H. (2014). Lateral stability simulation and analysis for wheel loaders based on the steady-state margin angle. Int. J. Model. Identif. Control.

[21] Nygren E., Sitaraman R.K., Sun J. (2010). The Akamai network: A platform for high-performance internet applications. ACM Sago’s Oper. Syst. Rev..

[22] De Vito S., Palma P.D., Ambrosino C., Massera E. (2011). Wireless sensor networks for distributed chemical sensing: Addressing power consumption limits with on-board intelligence. IEEE Sens. J..

[23] Lai Y.C., Jan S.S., Hsiao F.B. (2010). Development of a low-cost attitude and heading reference system using a three-axis rotating platform. Sensors.

[24] Madgwick S.O.H., Harrison A.J.L., Vaidyanathan R. Estimation of Imu and Marg Orientation Using a Gradient Descent Algorithm. Proceedings of the 2011 IEEE International Conference on Rehabilitation Robotics.

[25] Fourati H. (2015). Heterogeneous data fusion algorithm for pedestrian navigation via foot-mounted inertial measurement unit and complementary filter. IEEE Trans. Instrum. Meas..

[26] Carminati M., Ferrari G., Grassetti R., Sampietro M. (2011). Real-time data fusion and MEMS sensors fault detection in an aircraft emergency attitude unit based on Kalman filtering. IEEE Sens. J..

[27] Li X., Li Z. (2014). Vector-aided in-field calibration method for low-end mems gyros in attitude and heading reference systems. IEEE Trans. Instrum. Meas..

[28] Sipos M., Paces P., Rohac J., Novacek P. (2012). Analyses of tri-axial accelerometer calibration algorithms. IEEE Sens. J..

[29] DIN ISO (1984). Road Vehicles—Steady State Circular Test Procedure.

